# The influence of light microclimate on the lipid profile and associated transcripts of photosynthetically active grape berry seeds

**DOI:** 10.3389/fpls.2022.1022379

**Published:** 2023-01-04

**Authors:** Andreia Garrido, Artur Conde, Ric C. H. De Vos, Ana Cunha

**Affiliations:** ^1^Centre of Molecular and Environmental Biology (CBMA), Department of Biology, University of Minho, Braga, Portugal; ^2^Business Unit Bioscience, Wageningen Plant Research, Wageningen University and Research (Wageningen-UR), Wageningen, Netherlands

**Keywords:** light microclimate, grape seeds, lipids, oils, seed photosynthesis

## Abstract

Lipids and oils determine the quality and industrial value of grape seeds. Studies with legume seeds demonstrated the influence of light on lipid metabolism and its association with seed photosynthesis. Grape berry seeds are photosynthetically active till mature stage, but mostly during the green stage and veraison. The objective of this work was to compare the lipid profiles of seeds from white grape berries (cv. Alvarinho) growing at two contrasting light microclimates in the canopy (low and high light, LL and HL respectively), previously reported to have distinct photosynthetic competences. Berries were collected at three developmental stages (green, veraison and mature) and from both microclimates, and the seeds were analyzed for their lipid profiles in an untargeted manner using liquid chromatography coupled to high resolution mass spectrometry (LCMS). The seed lipid profiles differed greatly among berry developmental stages, and to a lesser extend between microclimates. The LL microclimate coincided with a higher relative levels of fatty acids specifically at mature stage, while the HL microclimate led to an up-regulation of ceramides at green stage and of triacylglycerols and glycerophospholipids at mature stage. The seed transcript levels of four key genes (*VvACCase1*, *VvΔ9FAD*, *VvFAD6* and *VvLOXO*) involved in fatty acid metabolism were analyzed using real-time qPCR. The lipoxygenase gene (*VvLOXO*) was down- and up-regulated by HL, as compared to LL, in seeds at green and veraison stages, respectively. These results suggest that seed photosynthesis may play distinct roles during seed growth and development, possibly by fueling different lipid pathways: at green stage mainly towards the accumulation of membrane-bound lipid species that are essential for cell growth and maintenance of the photosynthetic machinery itself; and at veraison and mature stages mainly towards storage lipids that contribute to the final quality of the grape seeds.

## 1 Introduction

Grape seeds are an important co-product from winemaking process (Teixeira et al., 2014). Their biochemical composition, namely the oil content, sets their economic value for several industries (Lucarini et al., 2018), such as nutraceutical, pharmacological and cosmetic industries (Garavaglia et al., 2016; Ananga et al., 2017).

Lipids are organic compounds formed by hydrocarbon chains of an hydrophobic or amphiphilic nature and have been divided into eight categories, following the classification proposed by LIPIDMAPS (http://www.lipidmaps.org/): fatty acyls (FA), which encompasses the free fatty acids (FFA), glycerolipids (GL), glycerophospholipids (GP), sphingolipids (SP), saccharolipids (SL), polyketides (PK) (derived from condensation of ketoacyl subunits); and sterols (ST) and prenols (PR) (derived from condensation of isoprene subunits) (Fahy et al., 2011). Furthermore, each of these categories includes distinct classes and sub-classes, increasing the complexity of this family of compounds.

The lipid biosynthetic pathway starts in the chloroplast, with the *de novo* synthesis of fatty acids, and continues in the cytosol, endoplasmic reticulum (ER) and oil bodies (Bates et al., 2013; LaBrant et al., 2018). The biosynthesis of fatty acids is a light-dependent reaction using the reducing power and adenosine triphosphate (ATP) generated during the photochemical phase of photosynthesis (Rawsthorne, 2002; Ye et al., 2020). Indeed, in the chloroplast, the ACCase enzyme initiates *de novo* fatty acids synthesis catalyzing the carboxylation of acetyl-CoA to malonyl-CoA in an ATP-dependent manner (Ye et al., 2020) (**Supplementary Figure 1**). Assembly of fatty acids occurs on acyl carrier protein (ACP) *via* a cycle of 4 reactions that elongate the acyl chain by 2 carbons each cycle. After 7 cycles, the saturated 16 carbon acyl-ACP can either be hydrolyzed by the FATB acyl-ACP thioesterase or further elongated by KASII (ketoacyl-ACP synthase II) to 18:0-ACP, which is then desaturated to 18:1-ACP by stearoyl-ACP desaturase (SAD) and hydrolyzed by the FATA thioesterase (Bates et al., 2013; LaBrant et al., 2018). Thus, Acyl-ACP thioesterases catalyze the hydrolysation of acyl-ACP to non-esterified (‘free’) fatty acids that are later activated to acyl-CoA by the long-chain acyl-CoA synthetase (LACSs) in the outer plastid envelope (i.e., in the cytosol) (Wang and Benning, 2012).

Part of the fatty acids synthesized in the envelope membranes of chloroplasts can be directly assembled into thylakoid lipids, while others can be exported to the ER for lipid assembly (Wang and Benning, 2012). The chloroplast-specific glycerolipids, i.e., monogalactosyldiacylglycerol (MGDG), digalactosyldiacylglycerol (DGDG), sulfoquinovosyldiacylglycerol (SQDG) and phosphatidylglycerol (PG), are particularly important to build a special hydrophobic matrix for pigment-protein complexes - the photosystems I and II - which are pivotal for the photochemical phase of photosynthesis (Kobayashi, 2016; LaBrant et al., 2018). Therefore, the fatty acids that derive from the *de novo synthesis* in the chloroplast, like palmitic acid (C16:0) and oleic acid (C18:1), are important precursors not only for the synthesis of all the membrane lipids, but also for the synthesis of polyunsaturated fatty acids (PUFAs), like linoleic acid (C18:2) and linolenic acid (C18:3) in the ER, and of storage lipids or oils (i.e., triacylglycerides or triacylglycerols, TAGs) (Bates et al., 2013). In the ER, the *de novo* assembly of TAG from glycerol-3-phosphate (G3P) and acyl-CoAs involves several steps of acylations, which are known as Kennedy pathway (Li-Beisson et al., 2013). Part of the phospholipids assembled *via* the ER pathway can return to plastids to serve as substrates for thylakoid lipid synthesis (Li-Beisson et al., 2013).

The desaturation of fatty acids occurs either in the chloroplast or in the ER (**Supplementary Figure 1**). In the stroma of chloroplast, the Δ9-fatty acid desaturase (Δ9FAD) is a critical enzyme in the synthesis of unsaturated fatty acids, having the capability to convert palmitic acid (C16:0) or stearic acid (C18:0) into palmitoleic acid (C16:1) or oleic acid (C18:1), respectively (Los and Murata, 1998). Fatty acid desaturase-6 (FAD6) and fatty acid desaturase-2 (FAD2) encode two desaturases that function in the plastid and in the ER, respectively, being responsible for converting oleic acid (C18:1) to linoleic acid (C18:2) (Mikkilineni and Rocheford, 2003; Dar et al., 2017). Then, other sequential fatty acid desaturases (i.e., FAD3 and FAD7/8, in the ER and chloroplast, respectively) play a key role in the synthesis of the PUFAs, like linolenic acid (C18:3) (Lee et al., 2012; He et al., 2020). In the cytoplasm, lipoxygenases (LOX) catalyze the oxygenation of PUFAs into oxylipins/hydroperoxides (Liavonchanka and Feussner, 2006; Mosblech et al., 2009), which are further converted by hydroperoxide lyases (HPL) to form smaller fatty acid fragments, including volatiles like alcohols and esters (**Supplementary Figure 1**).

In grape berries, both for winemaking production and for consumption, the level of PUFAs differs between tissues, being lowest in the pulp (28 mg *per* 100 g of dry weight - DW), followed by the skin (79 mg *per* 100 g DW) and highest in the seeds (7355 mg *per* 100 g DW) (Santos et al., 2011). Moreover, most of the seed lipids are TAGs, which are specifically accumulating in the endosperm (Pope et al., 1993). The TAG, or oil, content in grape seeds depends of grapevine cultivar and stage of seed maturity (Baydar and Akkurt, 2001; Rubio et al., 2009; Lachman et al., 2015). Seed development is divided in three distinct stages: stage I – morphogenesis (0 till 42 days after flowering, DAF); stage II – maturation or reserve accumulation (42 till 60 DAF); and stage III – desiccation (60 till 120 DAF) (Ristic and Iland, 2005; Angelovici et al., 2010). These three stages of seed development coincide with the stages of grape berry development, i.e., green, veraison and mature, respectively (Coombe, 1995). Seed oils are mainly accumulating during the maturation stage and then their levels decrease again, possibly due to partial breakdown along the seed desiccation process (Angelovici et al., 2010). In general, grape seed oil contents range between 10 to 20% (v/w), depending on the cultivar, and mostly consist of TAG rich in unsaturated fatty acids, like linoleic acid (C18:2, 60 to 70% of total fatty acid acyl chains) and oleic acid (C18:1, 19 to 27%) (Ohnishi et al., 1990; Baydar and Akkurt, 2001; Baydar et al., 2007). For this, grape seed oil is gaining popularity for the production of edible vegetable oil with presumed beneficial effects to human health (Yilmaz and Toledo, 2004; Matthäus, 2008).

Despite their inner core localization in fleshy fruits (e.g., apple, tomato and grape) and even in dehiscent fruits (e.g., pea pods), seeds can receive transmitted light, as reviewed by Aschan and Pfanz (2003). Moreover, from the early developmental stages to mature, seed coats contain chlorophyll and exhibit photosynthetic activity, as demonstrated in e.g. soybean seeds (Ruuska et al., 2004; Borisjuk et al., 2005; Rolletschek et al., 2005) and in grape seeds (Breia et al., 2013; Garrido et al., 2018; Garrido et al., 2019). It has been suggested that seed photosynthesis in legumes has an important role in lipid metabolism in several ways: 1) by producing nicotinamide adenine dinucleotide phosphate (NADPH) and ATP for the energetically expensive FA biosynthesis (Borisjuk et al., 2005; Rolletschek et al., 2005) and 2) by producing O_2_ to prevent anoxia inside the seeds, thus allowing to generate more energy and reductant power from mitochondrial respiratory process (Borisjuk and Rolletschek, 2009); and 3) through the Calvin-Benson cycle by re-fixing respiratory CO_2_, providing intermediates for metabolism (Ruuska et al., 2004). In this line, studies with legume seeds point to an effect of light on lipid metabolism *via* seed photosynthesis (Goffman et al., 2005; Allen et al., 2009) and Ruuska et al. (2004) showed that *Brassica napus* seeds in siliques exposed to light *in planta* produce more FAs than seeds from shaded siliques.

To the best of our knowledge, there are no studies on the influence of light microenvironment at the canopy level on grape seed lipid metabolism and its potential relation with seed photosynthesis. In addition, viticulture management practices that can influence the light microclimate in the canopy, as for instance those applied in grapevines to alleviate the impacts of climate changes including irrigation and spraying with sunlight reflecting minerals like kaolin, that can also interfere with the level of light that reach the berries, and thus affect the photosynthetic activity of its tissues, including seeds (Garrido et al., 2019). In the present work we evaluated the potential effects of light microclimate at the canopy level on the lipid profile of grape seeds from Alvarinho cultivar, by comparing seeds from shaded (low light) and fully-exposed (high light) berry clusters along their development, and to relate these effects to their differential photosynthetic activity (Garrido et al., 2019).

## 2 Material and methods

### 2.1 Grapevine field conditions and sampling

Grape berry samples were collected in 2018, from field-grown Alvarinho cultivar grapevines (*Vitis vinifera* L.) grafted onto 196-17 rootstock, in the organic vineyard Quinta Cova da Raposa in the Demarcated Region of Vinhos Verdes, Braga, Portugal (41°34’16.4”N 8°23’42.0”W) (Garrido et al., 2019). The vineyard is arranged in terraces along a granitic hillside with high drainage. The sector selected for the trial was located on a hill (300 m above sea level) with NW-SE orientation and the vineyard rows with a NE-SW orientation. The vine training system applied for this cultivar follows the settings of Sylvoz (Simple Ascending and Recumbent Cord), in which the parallel rows have a space of 3 m between them, each foot of the vines is spaced 3 m apart and the training system have a maximum height of 1.6 m. The climate is typically Mediterranean like, a warm-temperate climate with relatively high precipitation during winter but very low during the summer that are generally dry and hot (Kottek et al., 2006). The Minho viticulture region, in which the vineyard is localized, is generally climatically characterized by relatively high annual precipitation (1200 - 2400 mm) and relatively mild summers (summer mean temperatures ranging from 18 to 22 °C) (Fraga et al., 2014). During 2018, we reported the monthly values of temperature and precipitation, in supplementary material of Garrido et al., 2019. Overall, the growing season was atypical from a climatic point of view, with a relatively cold and extremely dry winter and a relatively cold spring with rainy periods during the vegetative growth of the grapevines.

Clusters with two contrasting light exposures were selected to harvest grape berries during their development: low light (LL) clusters that grew in the shaded inner zones of the canopy (approx. 50 μmol photons m^−2^ s^−1^ on average), and high light (HL) clusters that were exposed to direct sunlight most of the day (approx. 150 μmol photons m^−2^ s^−1^ on average) (more details in Garrido et al., 2019). Grape berries were randomly collected in the morning (9–10 a.m.) from both light microclimates and at three distinct developmental stages: Green [6 weeks after anthesis (WAA) or BBCH-75 - BBCH-scale used for grapes by Lorenz et al., 1994], Veraison (12 WAA, BBCH-83), and Mature (15 WAA, BBCH-89). At each developmental stage, grape berries were sampled as 3 (both veraison and mature) or 4 (green) biological replicates from each light microclimate condition, in which 1 replicate represented a mix of 15 to 20 berries, from 3 to 5 clusters from 6 to 8 plants growing in untreated vineyard plots (i.e., from the non-irrigated, non-kaolin control vines, as described in Garrido et al., 2019). The whole berries were immediately frozen in liquid nitrogen and stored at −80 °C. Later, the berries were broken with a slight impact of a pestle in a mortar (both pre-cooled with liquid nitrogen), which allowed us to isolate the seeds. The seeds were then ground to a fine powder and the samples (a total of 20) were freeze-dried (48 h) for lipid analysis. For the transcriptional analysis, for each condition, 3 independent subsamples of grape seeds were prepared from the biological replicates, resulting in a total of 18, which were stored till analysis or immediately used.

### 2.2 Lipid extraction, untargeted analysis by Liquid Chromatography Mass Spectrometry and data processing

The freeze-dried seed samples were extracted for lipidomics analysis, as described by Remmers et al. (2018). Quality control (QC) samples (*n* = 3), consisting of a mix of lyophilized material of grape seed samples, were simultaneously prepared and extracted, in order to estimate the overall analytical variation per detected compound. Ten mg of sample dry weight were extracted with 1.8 mL of chloroform/methanol (1:1, v/v), containing 0.1% (w/v) butylated hydroxytoluene (BHT) as antioxidant and 1 μM 1,2-didecanoyl-sn-glycero-3-phosphocholine (*Sigma*^®^ P7081) as internal standard. After two cycles of vortexing and 20 min on ice, the samples were centrifuged for 10 min at 16,100 x *g*, and the supernatants were transferred to new Eppendorf tubes. The organic solvent was evaporated during 1 h and 30 min in a Speed vac (*Savant^®^, SC100*). Prior to LCMS analysis, the obtained lipid fraction was dissolved in 200 μL ethanol (96%), vortexed, sonicated (5 min.) and again centrifuged for 10 min. at 16,100 x *g*. The supernatants (150 μL) were transferred to amber-coloured 2-mL HPLC vials with glass insert and sealed. The analysis was performed at LCMS system consisting of an Acquity UPLC (Waters), a Waters ACQUITY UPLC^®^ HSS T3 1.8 μm (1.0 x 100 mm) column and a LTQ-Orbitrap XL hybrid mass spectrometer (Thermo) in positive ionization mode, as described by Remmers et al. (2018).

Unbiased mass peak picking and alignment of the raw data sets from LCMS were carried out using MetAlign software (Lommen, 2009). Irreproducible individual mass signals (present in <3 samples) were filtered out using an in-house script called MetAlign Output Transformer (METOT) (Houshyani et al., 2012). The remaining mass peaks, including molecular ions, in-source adducts, fragments and their natural isotopes, were subsequently clustered using MSClust software into so-called reconstructed metabolites (centrotypes) (Tikunov et al., 2012), according to their corresponding retention time and peak intensity pattern across samples. In the final dataset, the total number of non-detected compounds, i.e. below the detection threshold of 5000 ion counts in any sample, was 4528 out of 11200 values in total. These non-detects were subsequently filtered out when not present in all 3 or 4 biological replicates of at least one sample group, leaving 376 lipid-soluble compounds. The relative intensities values of compounds were normalized by the internal standard. The values of the remaining non-detects (1493) were randomized between 45% and 55% of the detection threshold, i.e., between 2250 and 2750. The resulting spreadsheet (**Supplementary Table 1**) with the relative intensity of each metabolite in each sample was used for further statistical analyses.

### 2.3 RNA extraction and cDNA synthesis

RNA was extracted from all the 18 seed samples: 3 replicates x 2 microclimates x 3 developmental stages. The total RNA was purified according to Reid et al. (2006), with some adjustments. To 500 mg of frozen tissue, 3 mL of the extraction buffer containing 2% (w/v) of cetrimonium bromide (CTAB), 2% (w/v) of soluble polyvinylpyrrolidone (PVP) K-30, 300 mM of TRIS-HCl (pH 8.0), 25 mM of ethylenediamine tetraacetic acid (EDTA), 2 M of sodium chloride (NaCl), and 40 mM of dithiothreitol (DTT, mixed just prior to use) were added. Samples were incubated at 60 °C for 30 minutes and shaken every couple of minutes. After this, the mixtures were extracted twice with 3 mL of chloroform:isoamyl alcohol (24:1) followed by a centrifugation step at 3500 x *g* for 15 min at 4 °C. The aqueous fraction (1.5 mL) was mixed with 0.1 vol of 3 M NaOAc (pH 5.2) and 0.6 vol of isopropanol, and maintained at -80 °C for 30 min, after which the samples were centrifuged at 3500 x *g* for 30 min at 4 °C. The pellet was resuspended in 500 μL of plant RNA Lysis Solution from GeneJET Plant RNA Purification Mini Kit (Thermo Scientific^®^), following the manufacturer’s instructions. RNA concentration was determined in the Nanodrop (Thermo Fisher Scientific Inc.) and its integrity was assessed in a 1% agarose gel. Total RNA was further purified with DNase I Kit (Thermo Scientific^®^) to remove any contaminating DNA. First strand cDNA synthesis was synthesized from 1 μg of total RNA using the Xpert cDNA Synthesis Mastermix (Grisp^®^), following the manufacturers’ instructions.

### 2.4 Transcriptional analysis by real-time qPCR

Real-time qPCR was used for transcriptional analyses of target genes (**Supplementary Table 2**). The gene specific primer pairs used for each target or reference gene are listed in **Supplementary Table 2**. The primers of the target genes were designed using the software QuantPrime (Arvidsson et al., 2008). The analysis was performed with Xpert Fast SYBR (uni) Blue (Grisp^®^) using 1 μL cDNA (diluted 1:10 in ultra-pure distilled water) in a final reaction volume of 10 μL per well.

The transcriptional analyses were performed with an CFX96 Real-Time Detection System (Bio-Rad) using the following cycler conditions: polymerase was activated with an initial step of 3 min at 95 °C, the double strand denaturation occurred at 95 °C for 10 s, the annealing temperature was 55 °C during 20 s and the extension temperature was 72 °C during 20 s (amplification was performed using 40 cycles). Melting curve analysis was performed for specific gene amplification confirmation.

Actin 1 (*VvACT1*) and glyceraldehyde-3-phosphate dehydrogenase (*VvGAPDH*) were selected as reference genes, because these genes were proven to be highly stable and ideal for qPCR normalization purposes in grapevine (Reid et al., 2006). Additionally, for each qPCR analysis the actual stability of these target genes (i.e., no significant variation in their expression across sample groups) was validated by the M-values and coefficient of variance values calculated by CFX ManagerTM Software (Bio-Rad): for these parameters, the acceptable values for the stability should be less than 1 and 0.5, respectively (Hellemans et al., 2007). The expression values of target genes were normalized by the average of the expression of both reference genes, as described by Pfaffl (2001), and the results shown as arbitrary units (a.u.) of relative expression.

### 2.5 Statistical analysis

The on-line tool MetaboAnalyst (https://www.metaboanalyst.ca) was employed to compare the relative abundances of lipid-soluble compounds in the various grape seed samples (Xia et al., 2015). The compound spreadsheet was uploaded into this platform, and intensity data were Log_10_-transformed and scaled by the Pareto method (mean-centered and divided by the square root of standard deviation of each variable). Principal Component Analysis (PCA) was used as an unsupervised approach, to make a summary review of samples and to determine differences between developmental stages and between light microclimates. Analysis of Variance (ANOVA) test followed by *post hoc* multiple comparisons using the Tukey’s range test was employed to obtain the metabolites that contribute to the differences between developmental stages. In addition, a heatmap plot was made based on the 23 significant compounds according to ANOVA test, after adjudgment of the *p*-values using the Benjamini–Hochberg false discovery rate (FDR) correction.

In addition, to select those compounds that were most influenced by light microclimate, for each developmental stage a statistical analysis of the averages of the LL and HL groups was performed per compound separately, using Log_2_ transformed data and the Analysis ToolPak from Microsoft Excel^®^ (version: 16.0.1312721064) for performing Student’s *t*-tests. The list with significantly differing metabolites (*p* ≤ 0.05; after FDR correction of the *p*-values) and their respective fold change (FC) values (**Supplementary Tables 3****,** **4** **and** **5**) were considered for manual putative identification, based on the accurate mass of the most abundant ion in the mass cluster, which was presumed to represent the [M+H]^+^ or [M+NH4]^+^ adduct of the molecular ion, and the information available at LIPID MAPS^®^ Lipidomics Gateway (http://www.lipidmaps.org/). It is worth noting that we only focused on those significantly differing metabolites with the lowest *p*-values and for which the FC-values (i.e., size of the effect) was higher than the overall technical variation for that specific compound (as determined from the analytical quality control samples) (**Supplementary Table 1**).

The gene expression data was log(x+1) transformed to meet homogeneity of variances. Then, a two-way ANOVA was applied, followed by *post hoc* Bonferroni test whenever the factors (microclimate or developmental stage) had a significant effect (GraphPad Prism version 5.00 for Windows, GraphPad Software, La Jolla, California, USA).

## 3 Results and discussion

### 3.1 Seed lipid patterns during grape berry development

**Figure 1:**
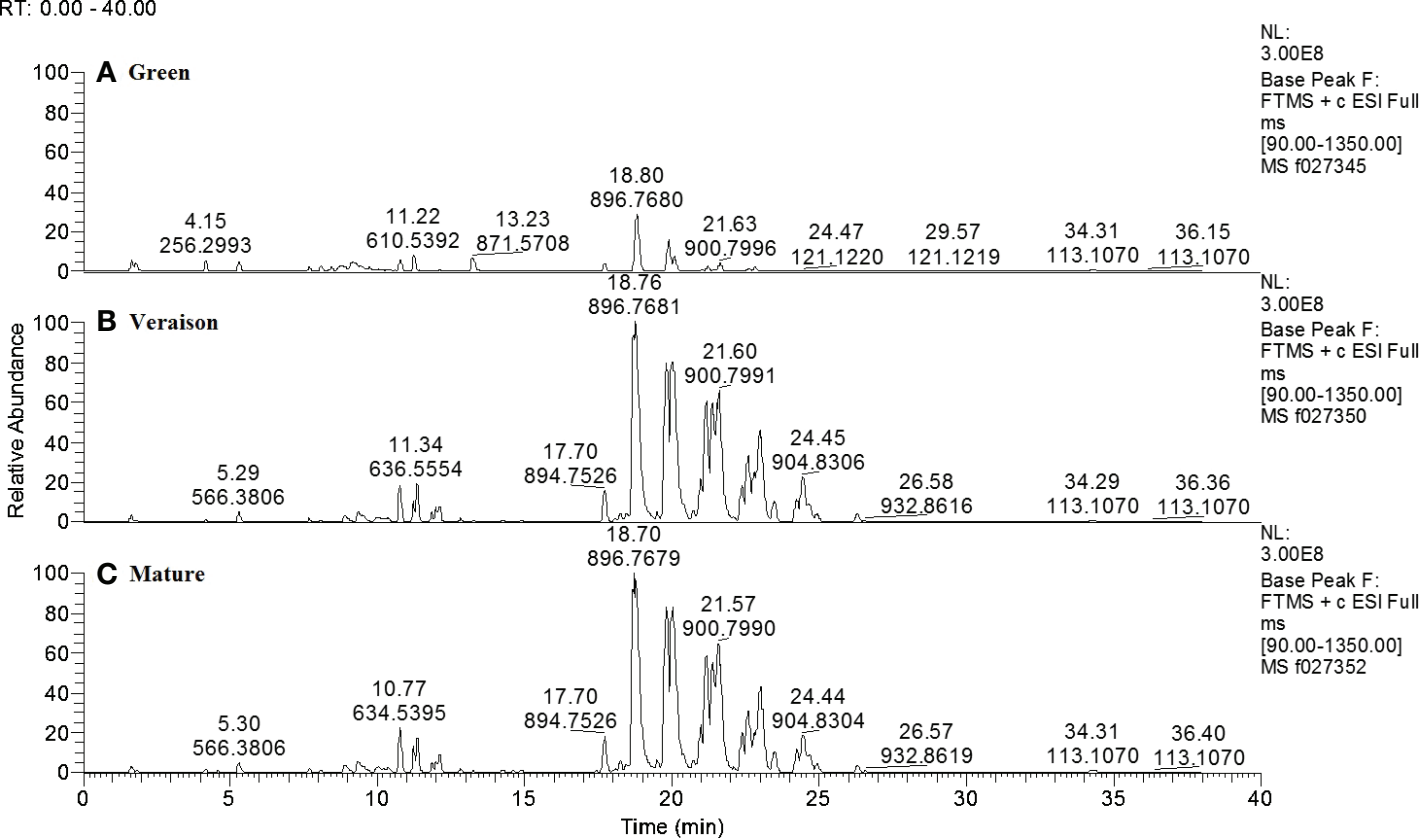
Representative LCMS chromatograms of lipid-soluble compounds in seeds from LL-grown Alvarinho berries at different developmental stages: green **(A)**, veraison **(B)** and mature **(C)**. Numbers above peaks represent, from top to bottom, the retention time (min) and accurate mass (m/z; positive ionization mode), respectively. The three chromatograms are in the same Y-scale. Annotations of compounds, if known, are provided in **Supplementary Table 1**.

Lipid profiles were assessed by high resolution Liquid Chromatography Mass Spectrometry (LCMS) of apolar chloroform-methanol extracts of seeds from berries at three developmental stages (green, veraison and mature) and growing in two distinct light microclimates in the grapevine canopy (low light – LL, shaded, and high light – HL, exposed to full sunlight, approx. 50 and 150 μmol photons m^−2^ s^−1^ on average, respectively). After untargeted data processing, a spreadsheet with the relative intensities of each detected compound in each sample was obtained, with a total of 376 putative lipid soluble compounds (**Supplementary Table 1**). The LCMS-lipidomics profiles of LL grown grape seeds (**Figure 1**) show that in green berries these seeds have an overall lower relative abundance of lipid compounds, per equal amount of dry weight, as compared to seeds from both veraison and mature berries; these later stages showed rather similar profiles. The same trend was observed for the lipidomics profiles of the HL grape seeds (data not shown). The highest peaks in the retention time window of 18 till 25 minutes correspond to the main lipid class of triradylglycerols and sub-class triacylglycerols, i.e. seed storage lipids.

**Figure 2:**
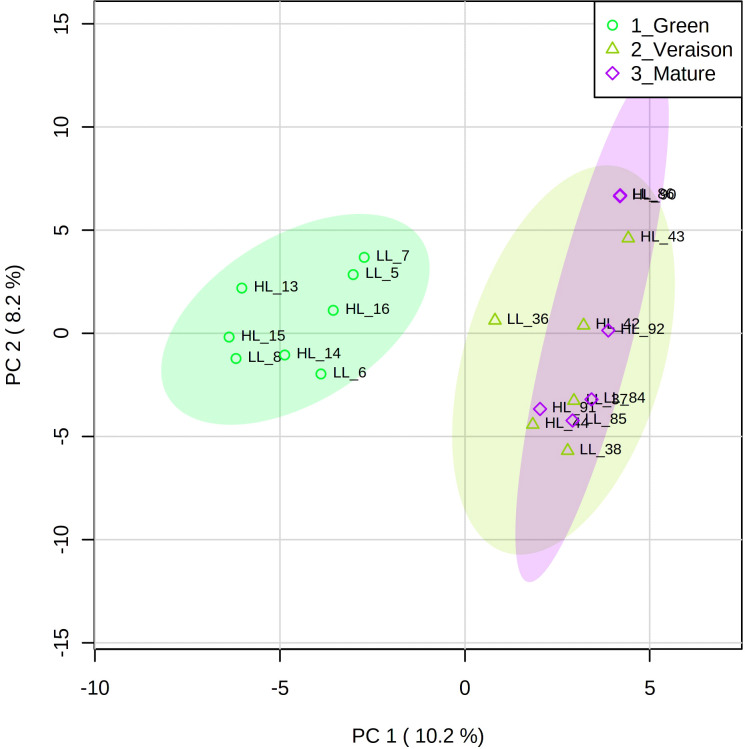
Principal component analysis (PCA) score plots of the lipidomics data for grape seeds from Alvarinho berries at three developmental stages (green, veraison, mature) and light microclimates (LL, low light; HL, high light). Colored ellipses represent 95% confidence interval (*n* = 4 for green stage and *n* = 3 for veraison and mature stages). The numbers next to the LL and HL indicate the sample identification.

Principal component analysis (PCA), an unsupervised multivariate analysis approach, was used to identify the main factors underlying the differentiation of grape seed samples as deduced from their lipidomics profiles (**Figure 2**). In this PCA plot, 18.4% of total variance is explained by the first two principal components. PC1 clearly distinguished the green stage from veraison and mature stages, supporting the observations from the chromatographic profiles (**Figure 1**). No conspicuous grouping related to microclimate was observed in this PC1-PC2 plot, neither in the PC2-PC3 plot (data not shown).

**Figure 3:**
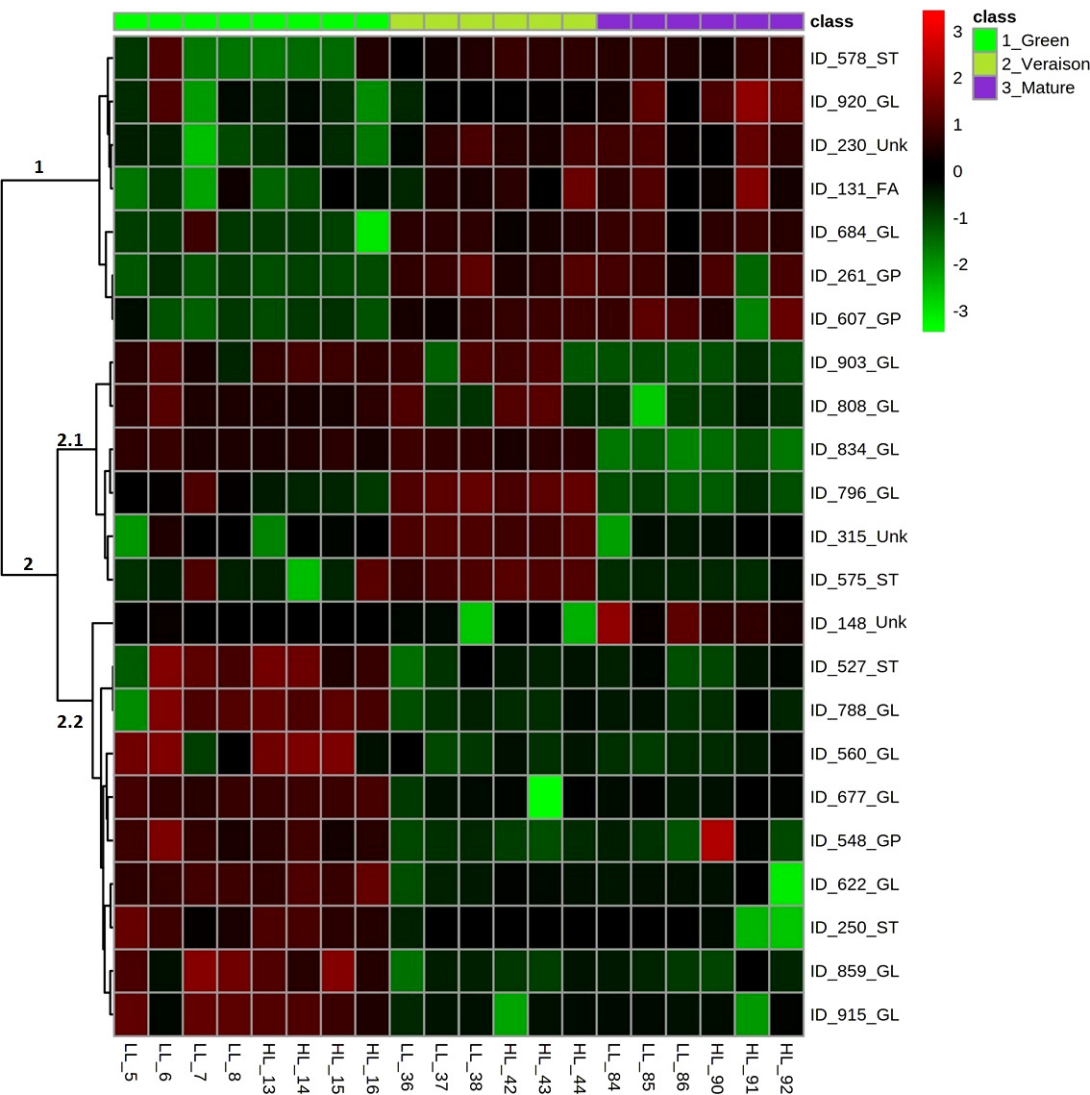
Heatmap with the 23 most significant seed lipids affected by grape berry developmental stages. On the left-hand side of the heatmap, the numbers represent the main clusters of the heatmap. On the right-hand side, the individual lipid compounds are represented by their LCMS number (e.g. ID_578) followed by the abbreviation of their lipid category: ST, Sterols; GL, Glycerolipids; FA, Fatty Acyls; GP, Glycerophospholipids; Unk, unknown. Samples and developmental stages are also indicated: the three developmental stages on top of the heatmap and ordered from left to right as green (light green color), veraison (darker green color) and mature (purple color), while the biological replicates for low light (LL) and high light (HL) are indicated at the bottom (*n* = 4 for green stage and *n* = 3 for veraison and mature stage each). Color scale on the top-tight of figure indicates the relative abundance of the lipid compounds in the samples, after logarithm data transformation (scale from -3 to +3, indicates a relatively low and high abundance, respectively).

The Analysis of Variance (ANOVA) test followed by *post hoc* multiple comparisons using the Tukey’s range test allowed us to find 23 lipid compounds responsible for the differences between developmental stages (data not shown). Based on this result, a heatmap plot based on these 23 metabolites ranked according to their ANOVA test results was constructed (**Figure 3**). Two main clusters (i.e., clusters 1 and 2) highlighted the differences between the green, veraison and mature stages. Cluster 1 represents a group of seed lipids with high relative abundance at veraison and mature stage berries, when compared with green stage, representing the following lipid categories: ST, FA, GL and GP. Cluster 2 encompasses seed lipids that had high relative abundance at green stage berries. Especially this cluster 2 appears divided into two other subgroups, coded 2.1 and 2.2, of which 2.1 contains lipids mainly from the GL category, which generally showed relative high abundances in both green and veraison stage berries, while 2.2 is represented by both ST, GL and GP categories and generally showed relative high abundances in the green stage berries only.

In our previous work we showed that grape seeds had high photosynthetic activity at both green and veraison stages, while slightly decreasing upon further berry development (Garrido et al., 2019). Thus, the relative high levels of GL present in seeds from only green (cluster 2.2) or both green and veraison stages (cluster 2.1) can be the reflex of the differences in seed photosynthesis between green/veraison and mature berries. It is reasonable to accept that tissues with greater photosynthetic competence are richer in thylakoid GL due to their crucial role in the photochemical phase of photosynthesis (Kobayashi, 2016). The relative high levels of GL in seeds from veraison and mature stage berries (cluster 1) may be associated with the accumulation of lipid reserves that mainly occurs at the stage II of seed development (Ristic and Iland, 2005). In addition, ST and GP are important components of the plasma membranes (Chen et al., 2009), and thus their relative high abundance in seeds of both green and veraison stage berries can be related with the high growth rate of cells in the seed of unripe berries (Ristic and Iland, 2005; Cadot et al., 2006).

### 3.2 Light microclimate effects on grape seed lipid metabolism: Approach to the potential roles of seed photosynthesis

Grape berry seeds are located in the core of an ovoid fruit, shielded not only by a pigmented exocarp but also by a compact mesocarp, thus receiving low amounts of transmitted sunlight. Aschan and Pfanz (2003) reported a value as low as 2.3% of the incident photon flux density. Therefore, and based on the light intensity that we previously measured at the grape berry surface level (Garrido et al., 2019), the seed samples used in this study received an estimated mean light intensity as low as 1.15 and 3.35 μmol photons m^-2^ s^-1^, at LL and HL microclimate, respectively, which nevertheless support distinct photosynthetic activities in the seeds (Garrido et al., 2019).

For each developmental stage, a Student’s *t*-test was performed to select those lipids that mostly contributed to the differences between LL and HL microclimates (*p* ≤ 0.05). The results show that out of the total of 376 compounds only 8, 10 and 20 metabolites were statistically different between LL and HL at green (**Table 1**), veraison (**Table 2**) and mature (**Table 3**) stage of development, respectively. For more details about the cluster ID’s metabolite number, see **Supplementary Table 1**.

**Table 1:** List of putatively annotated seed lipids with statistical differences between low light (LL) and high light (HL) microclimates at green stage of berry development (Student’s t-test, *p* ≤ 0.05).

Cluster ID	Fold Change (HL/LL)	*p* value	RT (min)	SIM mass	Mass Calculated	Adduct	Elemental Formula	Main class	LipidCategory	Sub-class
**412**	4.13	0.0085	11.64	784.6584	784.6579	[M+H]^+^	C_46_H_90_NO_6_P	Ceramides	SP	Ceramide 1-phosphates
**596**	1.50	0.0189	16.15	789.5931	789.5905	[M+NH_4_]^+^	C_46_H_78_NO_6_P	Glycerophosphocholines	GP	Dialkylglycerophosphocholines
**631**	0.69	0.0352	17.28	644.5961	–	–	–	Unknown	–	–
**915**	0.55	0.0315	23.88	890.8154	890.8171	[M+NH_4_]^+^	C_56_H_104_O_6_	Triradylglycerols	GL	Triacylglycerols
**78**	0.46	0.0375	4.17	256.2993	–	–	–	Unknown	–	–
**128**	0.40	0.0293	5.59	695.3903	–	–	–	Unknown	–	–
**726**	0.26	0.0150	19.53	698.6431	–	–	–	Unknown	–	–
**680**	0.22	0.0149	18.66	656.5960	–	–	–	Unknown	–	–

Metabolites are ordered by their fold change values (ratio HL/LL grapes) and the red-green patterns indicate the differences between the two light microclimates. For more details see **Supplementary Table 1**. Cluster ID – metabolite number. RT – chromatographic retention time. SIM mass – mz of putative molecular ion (highest signal in mass cluster). Lipid categories: SP, Sphingolipids; GP, Glycerophospholipids; GL, Glycerolipids.

**Table 2:** List of putatively annotated seed lipids with statistical differences between low light (LL) and high light (HL) microclimates at veraison stage of berry development (Student’s t-test, *p* ≤ 0.05).

Cluster ID	Fold Change (HL/LL)	*p* value	RT (min)	SIM mass	Mass Calculated	Adduct	Elemental Formula	Main class	LipidCategory	Sub-class
**101**	14.44	0.0077	4.90	283.2263	283.2268	[M+H]^+^	C_17_H_30_O_3_	Fatty Acids and Conjugates	FA	Hydroxy fatty acids
**480**	1.65	0.0312	13.05	968.7892	–	–	–	Unknown	–	–
**601**	1.44	0.0436	16.30	892.7373	892.7389	[M+NH_4_]^+^	C_57_H_94_O_6_	Triradylglycerols	GL	Triacylglycerols
**626**	1.42	0.0084	17.10	854.7215	854.7232	[M+NH_4_]^+^	C_54_H_92_O_6_	Triradylglycerols	GL	Triacylglycerols
**952**	1.29	0.0055	25.91	918.8462	918.8484	[M+NH_4_]^+^	C_58_H_108_O_6_	Triradylglycerols	GL	Triacylglycerols
**148**	1.27	0.0175	6.11	625.4297	–	–	–	Unknown	–	–
**780**	1.20	0.0348	20.29	926.8145	926.8171	[M+NH_4_]^+^	C_59_H_104_O_6_	Triradylglycerols	GL	Triacylglycerols
**183**	0.40	0.0221	6.96	801.5373	801.5389	[M+NH_4_]^+^	C_42_H_74_NO_10_P	Glycerophosphoserines	GP	Diacylglycerophosphoserines
**57**	0.21	0.0005	3.17	223.0961	223.0965	[M+H]^+^	C_12_H_14_O_4_	Fatty Acids and Conjugates	FA	Dicarboxylic acids
**713**	0.12	0.0031	19.06	228.9765	–	–	–	Unknown	–	–

Metabolites are ordered by their fold change values (ratio HL/LL grapes) and the red-green patterns indicate the differences between the two light microclimates. For more details see **Supplementary Table 1**. Cluster ID – metabolite number. RT – chromatographic retention time. SIM mass – mz of putative molecular ion (highest signal in mass cluster). Lipid categories: FA, Fatty Acyls; GL, Glycerolipids; GP, Glycerophospholipids.

**Table 3:** List of putatively annotated seed lipids with statistical differences between low light (LL) and high light (HL) microclimates at mature stage of berry development (Student’s t-test, *p* ≤ 0.05).

Cluster ID	Fold Change (HL/LL)	*p* value	RT (min)	SIM mass	MassCalculated	Adduct	Elemental Formula	Main class	LipidCategory	Sub-class
**727**	8.72	0.0003	19.60	925.8032	–	–	–	Unknown	–	–
**215**	6.16	0.0009	8.70	446.3982	446.3993	[M+NH_4_]^+^	C_29_H_48_O_2_	Sterols/Secosteroids	ST	Stigmasterols
**714**	5.50	0.0331	19.20	822.7535	822.7545	[M+NH_4_]^+^	C_51_H_96_O_6_	Triradylglycerols	GL	Triacylglycerols
**148**	5.11	0.0182	6.11	625.4297	–	–	–	Unknown	–	–
**171**	3.51	0.0273	6.71	796.5466	796.5487	[M+NH_4_]^+^	C_44_H_75_O_9_P	Glycerophosphoglycerols	GP	1-(1Z-alkenyl),2-acylglycerophosphoglycerols
**159**	3.13	0.0074	6.29	631.4166	–	–	–	Unknown	–	–
**185**	2.59	0.0386	7.20	772.5470	772.5487	[M+NH_4_]^+^	C_42_H_75_O_9_P	Glycerophosphoglycerols	GP	eg. 1-alkyl,2-acylglycerophosphoglycerols
**36**	2.40	0.0197	2.34	440.2481	–	–	–	Unknown	–	–
**192**	2.32	0.0112	7.18	730.5002	730.5041	[M+NH_4_]^+^	C46H_64_O6	Isoprenoids	PR	C40 isoprenoids (tetraterpenes)
**412**	2.13	0.0191	11.64	784.6584	784.6579	[M+H]^+^	C_46_H_90_NO_6_P	Ceramides	SP	Ceramide 1-phosphates
**123**	1.87	0.0341	5.46	678.4697	678.4704	[M+H]^+^	C_35_H_68_NO_9_P	Glycerophosphoserines	GP	1-(1Z-alkenyl),2-acylglycerophosphoserines
**85**	1.52	0.0031	4.40	316.2839	316.2846	[M+NH_4_]^+^	C_18_H_34_O_3_	Octadecanoids	FA	Other Octadecanoids
**956**	1.43	0.0386	26.33	880,8310	880.8328	[M+NH_4_]^+^	C_55_H_106_O_6_	Triradylglycerols	GL	Triacylglycerols
**953**	1.42	0.0414	26.10	830.7941	–	–		Unknown		–
**955**	1.22	0.0076	26.22	944.8617	944.8641	[M+NH_4_]^+^	C_60_H_110_O_6_	Triradylglycerols	GL	Triacylglycerols
**675**	1.19	0.0474	18.48	880.7157	880.7154	[M+H]^+^	C_52_H_98_NO_7_P	Glycerophosphocholines	GP	e.g: 1-alkyl,2-acylglycerophosphocholines
**952**	1.19	0.0169	25.91	918.8462	918.8484	[M+NH_4_]^+^	C_58_H_108_O_6_	Triradylglycerols	GL	Triacylglycerols
**53**	0.70	0.0065	3.02	228.1953	228.1958	[M+NH_4_]^+^	C_13_H_22_O_2_	Fatty Acids and Conjugates	FA	Unsaturated fatty acids
**136**	0.68	0.0042	5.81	354.3369	354.3367	[M+NH_4_]^+^ [M+H]^+^	C_22_H_40_O_2_	Fatty Acids and Conjugates Fatty amides	FA	Unsaturated fatty acids
**119**	0.59	0.0095	5.34	352.3203	352.3210	[M+NH_4_]^+^	C_22_H_38_O_2_	Fatty Acids and Conjugates	FA	Unsaturated fatty acids
**146**	0.51	0.0419	6.07	282.2785	282.2791	[M+NH_4_]^+^	C_18_H_32_O	Fatty aldehydes	FA	–
**110**	0.45	0.0032	5.20	415.3886	–	–	–	Unknown	–	–
**70**	0.17	0.0003	3.66	256.2266	256.2271	[M+NH_4_]^+^	C_15_H_26_O_2_	Fatty Acids and Conjugates	FA	Unsaturated fatty acids
**77**	0.09	0.0000	4.11	437.3728	-	–	–	Unknown	-	–

Metabolites are ordered by their fold change values (ratio HL/LL grapes) and the red-green patterns indicate the differences between the two light microclimates. For more details see **Supplementary Table 1**. Cluster ID – metabolite number. RT – chromatographic retention time. SIM mass – mz of putative molecular ion (highest signal in mass cluster). Lipid categories: GL, Glycerolipids; ST, Sterols; GP, Glycerophospholipids; PR, Prenols; SP, Sphingolipids; FA, Fatty Acyls.

At green stage, five lipids were influenced by the microclimate: one lipid was up-regulated (ID 412, FC HL/LL = 4.1), while four lipids were down-regulated by HL microclimate as compared with LL (ID 78, 128, 726, 680, all with FC < 0.5) (**Table 1**). The four lipids less accumulating in HL were putatively annotated as unknown, while the compound up-regulated by HL belongs to the main class of ceramides. Ceramides belong to the SP category, which are components of plasma membrane and endomembrane system, playing important roles in cell growth and differentiation and also operating as signaling mediators during plant responses to abiotic and biotic stresses (Ali et al., 2018; Huby et al., 2020; Liu et al., 2021). SP synthesis occurs in the endoplasmic reticulum, but it is directly linked to the lipid metabolic pathway in chloroplasts, where the synthesis of the initial precursor, i.e., the palmitoyl-CoA, takes place (Chen et al., 2009). To our knowledge, and although SP had already been associated with fleshy fruit development (Inês et al., 2018), this microclimate-related effect was for the first time reported. The lipids annotated as unknown could not yet be identified from the obtained putative molecular ion masses, thus future data-directed MSMS experiments to obtain mass spectra may help in their identification.

At veraison stage, one lipid from the main class of fatty acids (FA category) was up-regulated by HL, as compared to LL (ID 101, FC = 14) (**Table 2**). The relative high abundance of this hydroxy fatty acid in HL seeds is possibly the result of lipid oxidation processes, in line with our previous results from analyzing lipid peroxidation products using thiobarbituric acid which showed higher levels of these products in HL seeds compared to LL seeds (Garrido et al., 2021a). In addition, at veraison stage the HL grape seeds compared with LL ones had lower abundance in three lipids (FC < 0.5): ID 183 and ID 57, from the GP and the FA category, respectively, and the unknown ID 713.

In our previous work, we found that HL seeds from green berries exhibit more photosynthetic activity as compared to LL seeds at the same berry stage (Garrido et al., 2019). Moreover, during the ripening of grape berry and seeds a decrease in photosynthetic activity of seed outer integument from grapes grown in both microclimates was observed, but in particular for HL (Garrido et al., 2019). In this way, the higher levels of photosynthesis in HL green seeds can be pivotal to provide precursors, energy and reducing power for the synthesis of structural and regulatory lipids, like ceramides. In fact, previous works in a range of crop seeds verified that seed photosynthesis plays an indirect role in lipid metabolism, by providing O_2_ that is necessary for energy production (as reviewed by Borisjuk and Rolletschek, 2009; Tschiersch et al., 2011). In the case of grape seeds, the internal oxygen respiration peaks at around the beginning of ripening (i.e., around veraison stage) and then declines (Xiao et al., 2018). This way, seed photosynthesis can support the elevated demands for oxygen needed in lipid biosynthetic pathways, including those of ceramides and fatty acids, at early-mid (green and veraison) stages of grape seed development. In addition, the Calvin-Benson cycle intermediates of seed photosynthesis can be used for the production of primary substrates, such as acetyl-CoA necessary to fuel lipid biosynthesis. In particular, studies with carbon isotopes showed that high levels of incident light can influence the lipid metabolism in soybean seeds, by increasing intermediates generated by the Calvin-Benson cycle (Allen et al., 2009). Similarly, in rapeseed an increase in light intensity from 50 to 150 µmol m^-2^ s^-1^ enhanced oil biosynthesis (Goffman et al., 2005).

At mature stage the HL grape seeds, as compared with LL ones, contained a relative high level (FC > 2) of lipids from different categories, for instance: GL from the TAG sub-class (ID 714, FC = 5.4), ST (ID 215, FC = 6.1) and GP (ID 171, FC = 3.5) (**Table 3**). In fact, our results showed that HL mature grape seeds accumulated high levels of TAGs than LL ones and also several GP were up-regulated as compared to LL ones (**Table 3**).

Light intensity is an environmental factor associated with temperature, and thus the differential lipid profiles of grape seeds from HL and LL microclimates may also be related to the temperature differences. In wheat leaves it was shown that higher temperatures led to a decrease in the photosynthetic rate, an altered thylakoid membrane lipid composition, as well as other physiological changes (e.g., oxidative damage of cell organelles) (Djanaguiraman et al., 2018). Similarly, we previously showed that in grapes from the control conditions at veraison stage, the photosynthetic capacity of HL seed was lower compared to that of LL seed, but not in the green phase, where it was higher, and nor in the mature stage, where it was similar (Garrido et al., 2019). It is important to bear in mind that the latter study did not aim to evaluate the effect of temperature on the photosynthetic activity of grape berry tissues, however, HL grapes did exhibit higher mean temperatures than LL ones at all developmental stages (Garrido et al., 2019). Notwithstanding, the present lipidomics results showed that in the seeds of mature grapes several TAG species were significantly increased in HL as compared to LL condition, including mz-cluster ID’s 714, 956, 955 and 952 (**Table 3**), which is in line with the effect of high temperature stress on wheat leaves’ TAGs (Djanaguiraman et al., 2018).

In addition, the mature HL seeds had a lower abundance of unsaturated FA (e.g., ID 70 and 119) as compared to mature LL seeds (**Table 3**). Corroborating these results, in a study undertaken to examine the role of lipids in long-term acclimation to different growth light intensities in Arabidopsis leaves mutants, was shown that HL leaves (1000 µmol photons m^–2^ s^–1^) had lower rate of fatty acid synthesis in the chloroplast, but a higher fatty acid flux through the ER pathway of glycerolipid assembly, and thus, a higher triacylglycerol content, as compared with normal light grown leaves (200 µmol photons m^–2^ s^–1^) (Yu et al., 2021). In addition, the same authors showed that the plastid lipid biosynthetic pathway activity was significantly higher in leaves grown under LL (25 µmol photons m^–2^ s^–1^) compared with leaves grown under normal light (Yu et al., 2021). The authors suggested that the redirection of acyl chains through the ER pathway may be an adaptive benefit under HL conditions, because it increases energy consumption through glycerolipid synthesis (Yu et al., 2021). Indeed, this pathway requires additional ATP for both trafficking of fatty acids and complex lipids between the ER and the plastid and the ATP-dependent activation of free fatty acids at the outer chloroplast envelope (Li-Beisson et al., 2013). On the other hand, the increase of fatty acid flux through the plastid pathway reduces energy demand for lipid synthesis, which may confer a performance advantage under LL conditions (Yu et al., 2021). Considering this, the photosynthetic activity of grape seeds, may also play an important role in providing energy for these pathways.

In addition, and although the photochemical activity of seeds was lower at mature stage (Garrido et al., 2019), our recent data revealed that *VvRuBisCO* gene had similar expression level in seeds during all developmental stages and was up-regulated by HL (Garrido et al., 2021b). Therefore, this may suggest that the Calvin-Benson cycle (in its normal or partial operational form) can be active until later stages of seed development, especially in HL seeds, as an CO_2_ rescue mechanism, as proposed by Schwender et al. (2004) and Ruuska et al. (2004). Thus, the Calvin-Benson cycle may indirectly provide acetyl-CoA needed to *de novo* synthesis of FA in the chloroplast and hence fueling the synthesis of TAG, ST and GP in these mature seeds, ultimately potentially influencing seed and wine quality.

Overall, our results show that grape light microclimate influences the lipid profile of its seeds. Therefore, the photosynthesis (photochemical phase products and/or Calvin-Benson cycle) of the berry seeds may play an important physiological role in the biosynthesis of these lipids, in all developmental stages. As a result, these differences in seed lipid composition between HL and LL grapes may influence several biological functions at cell level and may also have impact on the quality of the grape seed by-products that can be used in the nutraceutical, pharmacological and cosmetic industries.

### 3.3 Transcriptional pattern of key genes involved in fatty acid metabolism

To the best of our knowledge there are no studies concerning the transcriptional analysis of genes coding enzymes involved in lipid metabolism of grape berry seeds. Therefore, the transcription levels of four key genes in fatty acid metabolism, i.e., acetyl-CoA carboxylase 1 (*VvACCase1*), stearoyl-[acyl-carrier-protein] *Δ*9-desaturase (*VvΔ9FAD*), fatty acid desaturase-6 (*VvFAD6*) and lipoxygenase (*VvLOXO*), were also evaluated. The enzymes encoded by these genes are not only involved in *de novo* synthesis of fatty acids, but also of PUFAs, lipids important for the thylakoid’s structure and other related to the production of flavor compounds, as shown in grape berry skin (Cramer et al., 2014).

**Figure 4:**
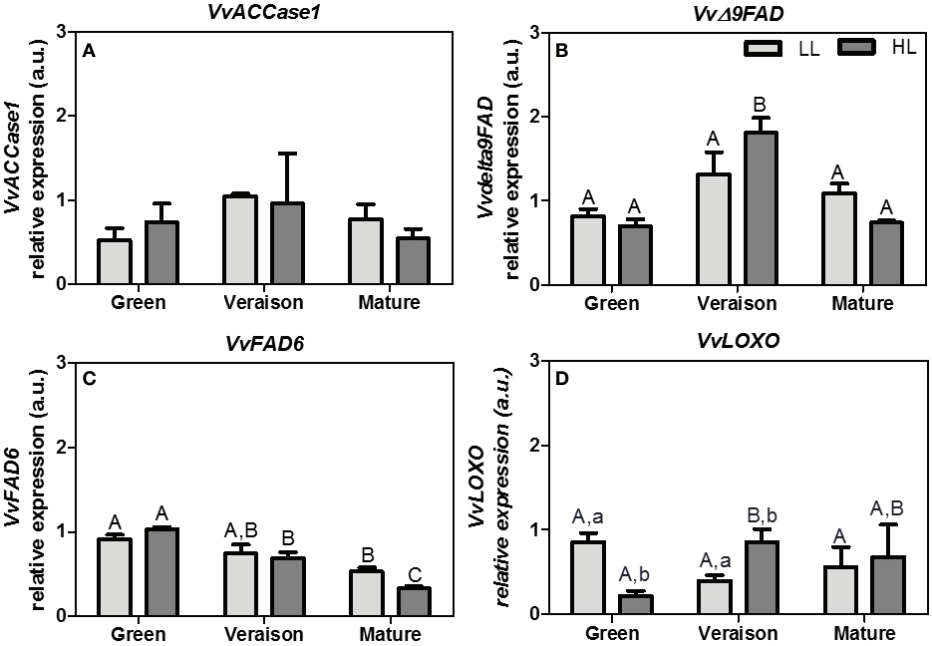
Relative expression arbitrary units (a.u.) of transcripts of: **(A)** acetyl-CoA carboxylase 1 (*VvACCase1*), **(B)** stearoyl-[acyl-carrier-protein] 9-desaturase (*VvΔ9FAD*), **(C)** fatty acid desaturase-6 (*VvFAD6*), and **(D)** lipoxygenase (*VvLOXO*). Gene expression analysis was performed by real-time qPCR in seeds from grapes grown at two light microclimates (low and high light, LL and HL, respectively) and at three developmental stages (green, veraison and mature). Expression levels are normalized to the mean expression of the reference genes *VvACT1* and *VvGAPDH*. Statistical analysis (two-way ANOVA, *p* ≤ 0.05, *n* = 3) was applied after data log(x+1) transformation. Statistical notation: capital letters refer to differences between developmental stages for the same microclimate, while lowercase letters refer to differences between microclimates for each stage. When the letters are omitted, it means that the respective factor did not have a significant effect.

Overall, and similar to the lipid profiles, the statistical differences between samples in gene expression were mainly related to the grape developmental stages (**Figure 4**). The expression of *Δ9FAD* gene peaked at veraison stage and then decreased at mature stage (**Figure 4B**). This pattern was quite similar to *VvACCase1*, the first gene of the fatty acid biosynthetic pathway (**Figure 4A**). These gene expression results are consistent with the fact that grape seeds at veraison stage accumulate high levels of TAG rich in unsaturated fatty acids (Rubio et al., 2009). Our results indicate that the expression of *VvFAD6* also decreased along seed maturation, in both microclimates (**Figure 4C**). Information concerning the expression pattern of these genes along seed development is relatively scarce in the literature. In soybean seeds the transcript levels of *FAD6* were relatively constant from young to mature stages (Heppard et al., 1996), in contrast to our results (**Figure 4C**). In addition, in flax seeds the relative expression of stearoyl-ACP desaturase (*Δ9FAD*) decreased along development (Fofana et al., 2006), while our results showed a peak at mid-developmental (veraison*)* stage (**Figure 4B**). Interestingly, a previous work with berry skin samples from the red grape variety Pinot Noir showed a decrease in *Δ9FAD* expression from post-veraison to harvest stage (Arita et al., 2017), corroborating our results in seeds from the white grape variety Alvarinho. Moreover, these authors also showed that the *VvFAD6* expression in berry skins from a white grape variety Koshu decreased along development, again in line with our results with seeds.

Of the 4 tested genes, the effect of microclimate was only statistically significant for *VvLOXO*: the HL microclimate first led to a down-regulation of *VvLOXO* expression in seeds at green stage and then to an up-regulation at veraison stage, as compared to the respective LL seeds (**Figure 4D**), as was also the tendency for *Δ9FAD* (**Figure 4B**). The fact that the light microclimate did not lead to a statistically significant difference in the transcripts abundance of fatty acid desaturase genes at any developmental stage (**Figures 4B, C**), together with the significant decrease of unsaturated fatty acid levels under HL conditions at mature stage (**Table 3**) suggests that fatty acid desaturases are regulated by post-transcriptional and/or post-translational regulatory mechanisms influenced by light. In this regard, in soybean photosynthetic cell suspensions, the plastidial gene ω3 fatty-acid desaturase (*FAD7*) has been shown to be post-transcriptionally regulated in response to light by specific changes in mRNA stability, but also regulated post-translationally at enzyme activity level (Collados et al., 2006).

Previously, Podolyan et al. (2010) characterized the enzymes and genes associated with the lipoxygenase pathway in a white grape variety (Sauvignon Blanc). They showed that *VvLOXO* was mostly expressed in seeds (75% of total expression) as compared to skin (21%) and pulp (4%). In plants, LOXs can be localized in cytoplasm and plastids, but also in other cellular organelles such as the vacuole, peroxisomes, lipid bodies, plasma membranes and microsomal membranes (as reviewed by Liavonchanka and Feussner, 2006). In grape berry, the presence of putative targeting peptides and *in silico* analysis of *VvLOXO* sequences suggested that this enzyme may be localized in chloroplasts (Podolyan et al., 2010).

Alterations in the LOX-HPL pathway may cause changes downstream, i.e. in resulting C6-alcohols and reducing thiols, and in this manner ultimately also in the final quality of the grapes and its wines. In this respect, the effects of light on LOX enzymes have been frequently studied (Xu et al., 2015; Joubert et al., 2016; Ju et al., 2016). For instance, Ju et al. (2016) showed that after removal of old leaves at veraison stage, resulting in more direct light on the berries, the LOX activity in grape skins was significantly higher than in the controls. In addition, the same authors also tested the fruit bagging treatment to decrease the light intensity received by the berries at veraison stage, which caused a significant decrease in LOX activity in grape skins of the treated berries as compared to the control. These results suggest that the activity of LOX in grapes is light dependent, and so viticulture practices that change the exposure of grape berries to daylight may have an impact on lipid metabolism. In accordance with these studies, our experiments showed that *VvLOXO* expression was up-regulated by HL microclimate in seeds from grapes at veraison stage, but also that this light regulation was dependent on the developmental stage (**Figure 4D**).

## 4 Integration and concluding remarks

The aim of the present research was to compare the lipid profiles of seeds from a white wine grape variety during the development of berries growing at two light different microclimates in the canopy of grapevine, low and high light, LL and HL, respectively. Since these contrasting microclimates are known to induced differential photosynthesis on the various grape berry tissues, including their seed (Garrido et al., 2019), we also intended to relate observed differences in seed lipid profiles to the differences in seed photosynthetic activities.

Overall, our results showed that the light microclimate influences seed lipid profiles in grapes at all developmental stages. At green stage, HL seeds had higher levels of ceramides, while at mature stage they contained higher relative levels of TAGs and GPs, as compared to LL seeds. Specifically, at mature stage, LL grape seeds had a relative higher abundance of the lipids from the category FA.

In our previous work we showed that the photosynthetic profile of both seeds from HL- and LL-growing grapes, decreased throughout seed development and maturation, but at different paces: the control HL grape seeds at green stage had significantly higher photosynthetic activity than LL ones, but for later stages of grape berry development (i.e., veraison and mature), these seeds had a higher reduction in the photosynthetic activity relatively to LL ones, being statistically lower at the veraison, but again similar at mature. Despite of this decrease till maturation, the maintenance of photosynthetic function till mature and the influence of the light microclimate on seed lipid and gene expression profile, suggests that the photosynthesis in grape seeds may play distinct roles and physiological functions at each developmental stage. For instance, at the green stage, seed photosynthesis can supply energy, reducing power and oxygen to fuel *de novo* biosynthesis of FA and ceramides, which are important components for an operational photosynthetic apparatus. In particular, the HL-induced increase in the relative abundance of ceramides may be associated with seed morphogenesis, cell growth and differentiation that occurs at early developmental stages (green stage). At veraison stage, the HL microclimate led to a higher abundance of seed FA, probably associated with the production of storage lipids, compared to LL. At the mature stage, the higher levels of TAG, ST and GP may be related with a still active Calvin-Benson cycle at the later stages of seed development (Garrido et al., 2021b), that can provide acetyl-CoA for synthesis of FA and related lipids.

In addition, the observed microclimate-dependent differences in the expression of key lipid-pathway genes in seeds also point to a possible role of berry photosynthesis in the final lipidome of this inner tissue. For instance, the HL-induced down-regulation of *VvLOXO* expression in seeds at green stage (**Figure 4D**) may translate into an inhibition of the biosynthesis of lipid oxidation-related products, e.g. lipid-derived volatiles, at the early phase of seed development, and potential carbon channeling into other important lipids (e.g. ceramides).

This study, to the best of our knowledge, provides the first comprehensive comparison of seed lipid profiles in grape berries growing in contrasting light microclimates. However, several questions remain to be answered, and thus this would be a fruitful area for further research, in order to explore the hypotheses addressed on the present work.

## Data availability statement

The original contributions presented in the study are included in the article/**Supplementary Material**. Further inquiries can be directed to the corresponding authors.

## Author contributions

Conceptualization, AG, RV, ACo and ACu; methodology, AG, RV, ACu and ACo; formal analysis, AG, RV, ACo and ACu; investigation, AG and ACu; resources, RV, ACo and ACu; writing—original draft preparation, AG; writing—review and editing, AG, RV, ACo and ACu; supervision, RV, ACo and ACu; project administration, ACu. All authors contributed to the article and approved the submitted version.
