# Supplementary material for: The influence of light microclimate on the lipid profile and associated transcripts of photosynthetically active grape berry seeds
Source: Front Plant Sci. 2023 Jan 4;13:1022379. doi: 10.3389/fpls.2022.1022379 (PMC9846335; doi:10.3389/fpls.2022.1022379)
Supplement: Supplementary file 1 [file Image_1.pdf]

Supplementary Material

The influence of light microclimate on the lipid profile and associated transcripts of photosynthetically active grape berry seeds

Andreia Garrido[1*], Artur Conde[1], Ric C. H. De Vos[2], Ana Cunha[1*]

[1]Centre of Molecular and Environmental Biology (CBMA), Department of Biology, University of Minho, Campus de Gualtar, 4710-057 Braga, Portugal.

[2]Business Unit Bioscience, Wageningen Plant Research, Wageningen University and Research (Wageningen-UR), PO Box 16, 6700 AA Wageningen, The Netherlands.

*** Correspondence:**
Andreia Garrido; Ana Cunha.
andreiagarrido@sapo.pt; accunha@bio.uminho.pt

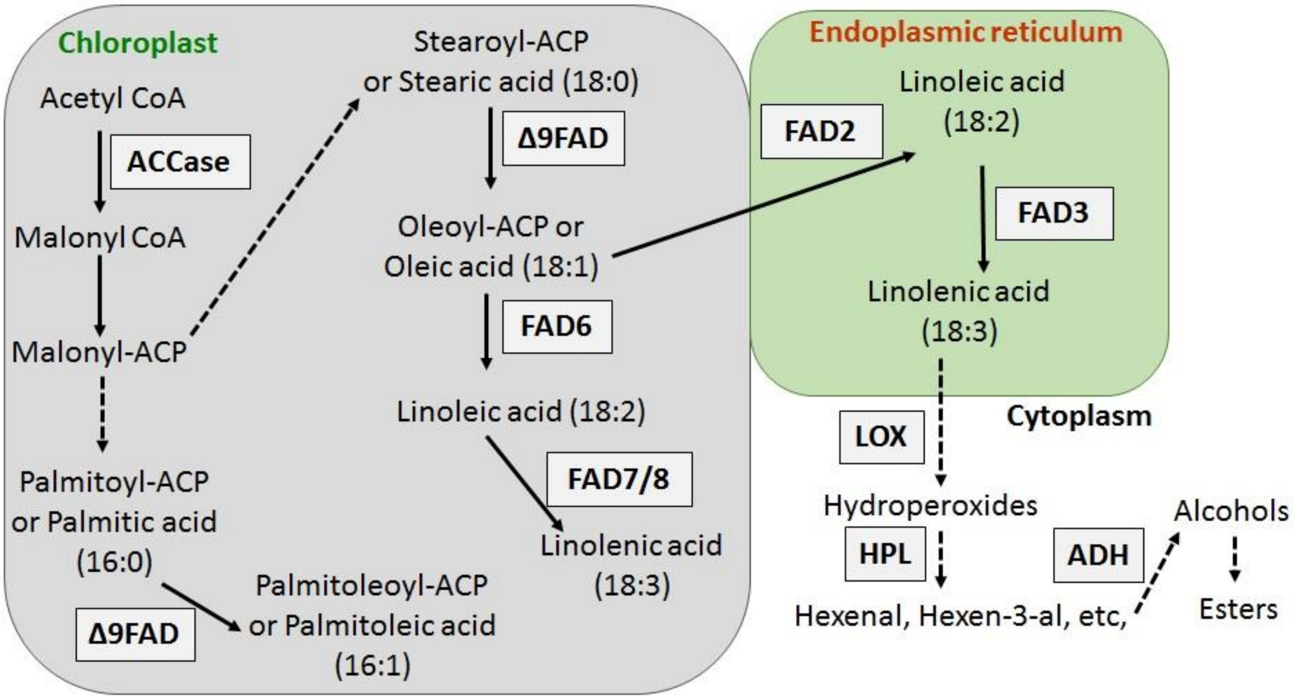

Supplementary Figure 1. Simplified metabolomic pathway of fatty acids synthesis and oxidation of polyunsaturated fatty acids into the production of flavor compounds like alcohols and esters. Abbreviations: Δ9FAD, stearoyl-[acyl-carrier-protein] Δ9-desaturase; ACCase, acetyl-CoA carboxylase; ADH, alcohol dehydrogenase, ACP, acyl carrier protein, FAD, fatty acid desaturase; HPL, hydroperoxide lyase; LOX, lipoxygenase. Adapted from Los and Murata (1998), Bates et al. (2013), Cramer et al. (2014), Dar et al. (2017) and He et al. (2020).
