# Supplementary material for: The influence of light microclimate on the lipid profile and associated transcripts of photosynthetically active grape berry seeds
Source: Front Plant Sci. 2023 Jan 4;13:1022379. doi: 10.3389/fpls.2022.1022379 (PMC9846335; doi:10.3389/fpls.2022.1022379)
Supplement: Supplementary file 3 [file Table_2.pdf]

Supplementary Material

The influence of light microclimate on the lipid profile and associated transcripts of photosynthetically active grape berry seeds

Andreia Garrido[1*], Artur Conde[1], Ric C. H. De Vos[2], Ana Cunha[1*]

[1]Centre of Molecular and Environmental Biology (CBMA), Department of Biology, University of Minho, Campus de Gualtar, 4710-057 Braga, Portugal.

[2]Business Unit Bioscience, Wageningen Plant Research, Wageningen University and Research (Wageningen-UR), PO Box 16, 6700 AA Wageningen, The Netherlands.

*** Correspondence:**
Andreia Garrido; Ana Cunha.
andreiagarrido@sapo.pt; accunha@bio.uminho.pt

Supplementary Table 2. Forward (F) and reverse (R) primers used for gene expression analysis by real-time PCR. Sequences accession numbers were obtained through Grape Genome Browser 12x.

Gene	Primers	Reference
***VvACT1* (reference gene)**	F: 5′-GTGCCTGCCATGTATGTTGCC-3′	Reid et al. (2006)
	R: 5′-GCAAGGTCAAGACGAAGGATA-3′	
***VvGAPDH* (reference gene)**	F: 5′-CACGGTCAGTGGAAGCATCAT-3′	Gainza-Cortés et al. (2012)
	R: 5′-CCTTGTCAGTGAACACACCAG-3′	
VvACCase1	F: 5′-TCCTTCAGGCAGGATCAACCATAC-3′	Cramer et al. (2014)
	R: 5′-TGATCCGACTGTCCACAACAACC-3′	
VvΔ9FAD	F: 5′-CTTGATGGGGTGAGAGATGAGA-3′	Arita et al. (2017)
	R: 5′-ACCCAACCAGAAAGATAGAGATAGG-3′	
VvFAD6	F: 5′-CAATTCGGCCTTGGATGTCT-3′	
	R: 5′-TGCCAAACTTATCTTCACCCTCTT-3′	
VvLOXO	F: 5'-TTCCACCCACTCGCCTGATG-3'	Podolyan et al. (2010)
	R: 5'-GCACCGCACCTGTTTCTTCG-3'	

Reference

Gainza-Cortés, F., Pérez-Dïaz, R., Pérez-Castro, R., Tapia, J., Casaretto, J. A., González, S., et al. (2012). Characterization of a putative grapevine Zn transporter, VvZIP3, suggests its involvement in early reproductive development in *Vitis vinifera* L. *BMC Plant Biol.* 12, 111. doi: 10.1186/1471-2229-12-111
